# Sublethal Effects of Imidacloprid on Fecundity, Apoptosis and Virus Transmission in the Small Brown Planthopper *Laodelphax striatellus*

**DOI:** 10.3390/insects12121131

**Published:** 2021-12-17

**Authors:** Yuanyuan Zhang, Gang Xu, Yu Jiang, Chao Ma, Guoqing Yang

**Affiliations:** 1 College of Horticulture and Plant Protection, Yangzhou University, Yangzhou 225009, China; zhangyuanyuan182@126.com (Y.Z.); jiangyu1126@outlook.com (Y.J.); machaoyzu@163.com (C.M.); 2 Jiangsu Co-Innovation Center for Modern Production Technology of Grain Crops, Yangzhou University, Yangzhou 225009, China; 3 Joint International Research Laboratory of Agriculture and Agri-Product Safety of the Ministry of Education of China, Yangzhou University, Yangzhou 225009, China

**Keywords:** imidacloprid, *Laodelphax striatellus*, fecundity, apoptosis, virus transmission

## Abstract

**Simple Summary:**

The small brown planthopper (SBPH) *Laodelphax striatellus* is an economically important pest in Asia, especially in China. Imidacloprid, a neonicotinoid insecticide, is commonly applied in rice fields to control the planthoppers. However, the widespread application of imidacloprid also has led to the development of resistance and to other potentially negative effects on crop protection. The sublethal effects of imidacloprid have been reported in many insects. Here, we investigated the potential effects of different sublethal concentrations of imidacloprid on SBPH and found that imidacloprid could affect the fecundity, apoptosis and virus transmission in the viruliferous SBPH. The results indicated that sublethal concentrations of imidacloprid may increase the fecundity of SBPH and the impact of insecticides on the transmission of plant viruses by insects should be considered when insecticides are applied to manage insect pests.

**Abstract:**

*Laodelphax striatellus* damages plants directly through sucking plant sap and indirectly as a vector of rice stripe virus (RSV), resulting in serious losses of rice yield. It is one of the most destructive insects of rice in East Asia. Insecticides are primarily used for pest management, but the sublethal concentrations of insecticides may benefit several insects. The present research attempted to explore the effects of sublethal concentrations of imidacloprid on the fecundity, apoptosis and RSV transmission in the viruliferous SBPH. The results showed that the fecundity of SBPH was significantly increased after treatment with the LC_10_ dose of imidacloprid, while the LC_30_ dose of imidacloprid reduced the fecundity compared with the control. To further investigate the underlying mechanism of increased fecundity after exposure to the LC_10_ dose of imidacloprid, we examined the expression levels of vitellogenin (*Vg*), *Vg* receptor (*VgR*) and caspases in the ovaries of SBPH, and observed the apoptosis by terminal deoxynucleotidyl transferase (TDT)-mediated dUTP-digoxigenin nick end labeling (TUNEL). qRT-PCR results indicated that the expression levels of *Vg*, *VgR* and four caspase genes were all significantly increased by the LC_10_ dose of imidacloprid, and TUNEL assays suggested that the frequency of apoptosis was significantly higher in the SBPH treated by the LC_10_ dose of imidacloprid, suggesting a potential correlation between the increased fecundity and the apoptosis of SBPH ovarioles. Additionally, the expression levels of *RNA3* and capsid protein (*CP*) were both increased significantly by the LC_10_ dose of imidacloprid, whereas were decreased by the LC_30_ dose of imidacloprid compared to the control. Therefore, this study clarifies the mechanisms of sublethal effects of imidacloprid on viruliferous SBPH and could be used to optimize pest control strategies.

## 1. Introduction

The small brown planthopper (SBPH), *Laodelphax striatellus* (Fallén) (Hemiptera: Delphacidae) is one of the most serious pests in East Asian rice fields, which not only causes direct damage via sucking rice sap and laying its eggs into rice tissues, but also acts as a viral vector that transmits rice stripe virus (RSV) [1,2,3]. RSV is a typical persistent-propagative plant virus, which is effectively transmitted by SBPH in a persistent and circulative-propagative manner [3,4]. RSV is ingested when SBPH feed on the rice infected by RSV, and it firstly establishes infection in the midgut epithelium, then spreads to midgut visceral muscle tissues, and ultimately enters the salivary glands, ovaries and other systemic tissues of SBPH under the transportation of hemolymph [3]. The virus entering the salivary gland is transmitted to healthy rice plants together with saliva to complete the horizontal transmission [5]. The virus binds to vitellogenin (*Vg*), a precursor of vitellin in the hemolymph, attaches to the nurse cells via *Vg* receptor (*VgR*)-mediated endocytosis and then is transovarially transmitted to offspring for vertical transmission in SBPH [4,6,7].

The management of SBPH mainly depends on chemical insecticides, including pymetrozine, imidacloprid, buprofezin, chlorpyrifos, thiamethoxam, and nitenpyram [8]. However, widespread application of these insecticides has resulted in the development of resistance and resurgence [9]. Besides, the application of neonicotinoid insecticides such as imidacloprid has caused damages to important pollinators such as bees [10]. Some studies demonstrated that SBPH has developed resistance to imidacloprid, buprofezin and chlorpyrifos [8]. Additionally, the study showed that the application of validamycin and triazophos increases the fecundity of SBPH in rice [11]. In the field, insecticides degrade over time or via wind, photolysis and hydrolysis, the concentration of insecticides initially used to control insects decrease until they reach the sublethal levels, resulting in sublethal effects on insects [12,13]. The biological performances and physiological processes of insects would be modified when exposed to sublethal doses of insecticides [14,15].

Imidacloprid, a systemic neonicotinoid, acts as on nicotinic acetylcholine receptor via disrupting the neuronal cholinergic signal transduction, is widely used against Hemiptera insects that damage plants via sucking sap [10,16]. Due to its low toxicity to mammals and long-acting to target insects, imidacloprid has been used worldwide to control sucking insect such as rice planthoppers [17,18]. Sublethal effects of imidacloprid have been reported in many insects, e.g., affecting biological performances, changing enzyme activities or influencing gene expression [19,20,21]. Several studies suggested that the sublethal concentrations of imidacloprid negatively affected fecundity in exposed insects, such as *Bemisia tabaci* [22], *Sogatella furcifera* [23], *Rhopalosiphum padi* [20], *Harmonia axyridis* [24], and *Ceratomegilla undecimnotata* [25]. However, exposure to low concentrations of imidacloprid could benefit several insects, including *Aphis glycines* [26] and *Myzus persicae* [27]. Meanwhile, *Vg* and *VgR* are essential proteins related to the fecundity of females in insects, and mRNA expressions of *Vg* and *VgR* could be influenced by sublethal exposure to imidacloprid [28,29].

Apoptosis, a programmed cell death process, which eliminates extraneous cells in vertebrates and invertebrates, maintains the normal development and cell homeostasis to reply to external stimuli [30,31,32]. The enzymes caspases are the main effectors of apoptosis pathway, which are divided into initiators and effectors according to its biological functions. Once the initiator caspases are activated, they will initiate the processes of apoptosis, and then the effector caspases will cleave various cell substrates, eventually leading to apoptosis [33,34,35]. In SBPH, four caspase genes have been identified, including *caspase-Nc*, *caspase-8*, *caspase-1a* and *caspase-1c* [36]. Much external stress stimuli, including pesticides, could induce apoptosis in insects [37]. Previous studies showed that a variety of pesticides can induce apoptosis in the salivary glands, midguts, ovaries and other tissues of the bees [37,38,39]. After exposure to imidacloprid, apoptosis was stimulated in the honey bees [40,41] and bugs [42,43]. Apoptosis is closely related to the oogenesis of insects. Nurse cells in the ovary provide nutrients for the growing of oocytes, and the apoptosis of these nurse cells is crucial to the maturation of oocytes [44]. Notably, the fecundity of SBPH was increased by *Wolbachia* infection, which appeared to be at least partly regulated through caspase-mediated apoptosis [45]. In addition to fecundity, apoptosis is also closely related to the virus transmission of vector insects. A recent study showed that infection with tomato yellow leaf curl virus induced the apoptosis pathway in *B. tabaci*, and the virus-induced apoptosis could increase viral accumulation and transmission in vectors [46].

Though sublethal effects of imidacloprid have been investigated in many insects, the effects of sublethal doses of imidacloprid on the viruliferous SBPH remains unclear. In the present study, we assessed sublethal effects of imidacloprid on the fecundity, apoptosis, and RSV transmission in SBPH. These results will contribute to understanding sublethal doses of imidacloprid-mediated effects on SBPH and provide new insights on pest control.

## 2. Materials and Methods

### 2.1. Insects

The SBPH strains were derived from a field population in Yangzhou, Jiangsu Province, China, and continuously maintained in the laboratory. Rice seedlings Wuyujing 3, supplied for SBPH, were grown in the soil at 26 ± 1 °C, 80 ± 5% RH with a 16:8 h (light: dark) photoperiod in an incubator. SBPH were fed by fresh rice seedlings (5–6 cm high) in a glass beaker [11].

The viruliferous (RSV-infected) and non-viruliferous (RSV-free) SBPH were used to screen for RSV. When SBPH emerged about 24 h later, each pair of SBPH was mated and put into the glass tube with fresh rice seedlings. Each pair was kept for 72 h to ensure the fertilization of the female, and then the female was raised individually for oviposition. A dot immunobinding assay (DIBA) [47] was applied to detect RSV when the females died. When the female was RSV-infected, its offspring were regarded as viruliferous and then used for subsequent experiments.

### 2.2. Bioassays

The rice seedling immersion method [11] was applied to evaluate the susceptibility of viruliferous planthoppers to imidacloprid (95%, Jiangsu Changqing Agrochemical Co., Ltd., Yangzhou, China). Imidacloprid was dissolved with acetone, and diluted using 0.05% Tween-80 emulsifying water, then the preparation was diluted directly with water, five serial dilutions (100, 50, 25, 12.5, and 6.25 mg/L) were made. Tap water without organic solvent and surfactant was performed as a control. Rice seedlings were immersed in the imidacloprid solutions for 30 s, their roots were enclosed with cotton to moisturize and then put in the individual tube (3 cm diameter, 20 cm depth) with 5 mL nutrient solution at the bottom after being air dried. Thirty third instar nymphs of SBPH were then transferred into the tubes, and each treatment was replicated three times. After 72 h, we counted the number of dead insects.

To evaluate the effects of different sublethal doses (LC_10_, LC_20_, and LC_30_) of imidacloprid on the viruliferous SBPH, approximately 500 third instar nymphs of SBPH were transferred to the glass beakers with the rice seedlings exposed to sublethal concentrations (LC_10_, LC_20_, and LC_30_) of imidacloprid, respectively. After 72 h exposure, the surviving insects were transferred to fresh rice seedlings for subsequent experiments.

### 2.3. Effects of Imidacloprid on Fecundity in SBPH

Approximately 24 h after emergence, each pair of SBPH exposed to different sublethal doses (LC_10_, LC_20_, and LC_30_) of imidacloprid was transferred to fresh rice seedlings in glass tubes. Tap water without organic solvent and surfactant was used as a control. Each treatment contained 30 pairs of SBPH. We changed rice seedlings every two days and used the binocular stereomicroscope to count the eggs. The number of eggs were recorded until the female died. If the male died, a new male was added until the experiment ended.

### 2.4. Quantitative Real-Time Polymerase Chain Reaction (qRT-PCR)

After treatments with different concentrations of imidacloprid, 30 ovaries from 4-days old female SBPH were dissected. Each treatment had three biological replicates. Tissues were stored with the TRIzol solution (Invitrogen, Waltham, MA, USA) at −70 °C after dissection, and Tissuelyser II (Qiagen, Hilden, Germany) was applied to homogenize the samples to extract total RNA according to the manufacturer’s protocols. The NanoDrop 2000 spectrophotometer (Thermo Fisher, Waltham, MA, USA) was used to measure the RNA concentrations and purity.

PrimeScript™ RT Master Mix (Takara, Tokyo, Japan) was used to transcribe the total RNA (1 μg) into cDNA for qRT-PCR analysis, and the Primer 3 (http://bioinfo.ut.ee/primer3-0.4.0/, accessed on 15 November 2021) was performed to design the primers for qRT-PCR (Table S1). The qRT-PCR reaction was run in the CFX Connect^TM^ Real-Time System (Bio-Rad, Hercules, CA, USA), with the final volume of 20 µL, containing 2 μL of template cDNA, 10 μL of ChamQ^TM^ SYBR qPCR Master Mix (Vazyme, Nanjing, China), 0.8 μL of each primer (10 μM) and 6.4 μL of ddH_2_O. The procedure of qPCR was 50 °C for 2 min, 95 °C for 5 min, followed by 40 cycles of 95 °C for 15 s, 60 °C for 45 s with the step of melt curve dissociation. The relative expression level of triplicate samples was calculated using the 2^−ΔΔCT^ method [48], and the expression levels of target genes were normalized to the reference gene *β*-*actin* [49].

### 2.5. Effects of Imidacloprid on Vertical Transmission of RSV by SBPH

Forty mating pairs treated by different concentrations of imidacloprid were prepared for each treatment. Tap water without organic solvent and surfactant was used as a control. Each pair was mated for 72 h to ensure that the female was fertilized. The DIBA [47] was used to detect whether the females were RSV-infected, after they died. The offspring of viruliferous females were regarded as viruliferous individuals, while the offspring of non-viruliferous females were removed. When the nymph hatched about 24 h later, they were transferred into glass cups containing new rice seedlings. When the nymphs grew to the third instar, they were used for RSV detection, and the vertical transmission rate refers to the proportion of viruliferous individuals in the total number of offspring [50].

### 2.6. TUNEL Assay

The greatest fecundity of SBPH appears at 4 days post-emergence, while after 10 days almost all reproductive ability is lost [45]. Therefore, after imidacloprid treatment, the ovaries from viruliferous SBPH at 4 and 10 days after emergence were dissected for observing the apoptosis via TUNEL staining. TUNEL preferentially labels relatively late apoptotic cells during apoptosis and helps to independently identify apoptotic cells in ovaries [51]. According to the manufacturer’s instructions [45] with a little modification, the TUNEL assay and experimental procedures were performed with the Dead End^TM^ Fluorometric TUNEL System kit (Promega, Madison, WI, USA). For apoptotic cell death analyses, ovaries of SBPH fed on rice seedlings treated with tap water and LC_10_ of imidacloprid were dissected. After hybridization, the samples were washed two times and then fixed on a glass slide using the DAPI-containing mounting Vectashield (Vector Laboratories, Burlingame, CA, USA). Finally, an ultra-high resolution laser scanning microscope (Leica, Heidelberg, Germany) was applied to analyze the samples. Cells stained with TUNEL and DAPI fluorescences were regarded as apoptotic cells.

Due to the difficulty of maintaining integrity of ovaries under staining, we counted the TUNEL-positive tropharia and expressed them as a percentage of the examined tropharia. The frequency of apoptotic tropharia was compared between the control and treated females.

### 2.7. Statistical Analysis

The probabilistic unit (Probit) regression in SPSS version 16.0 software (SPSS Inc., Chicago, IL, USA) was used to determine the bioassay results of imidacloprid, including LC_10_ to LC_30_ values with 95% confidence limits. GraphPad Prism version 8.0.0 (GraphPad Software, San Diego, CA, USA) was used to analyze and visualize the data. The data of vertical transmission rates and TUNEL-positive rates were analyzed by a Chi-squared (χ^2^) test. The differences between the two groups were compared using the Student’s *t*-test.

## 3. Results

### 3.1. Effects of Imidacloprid on the Fecundity of SBPH

**Figure 1:**
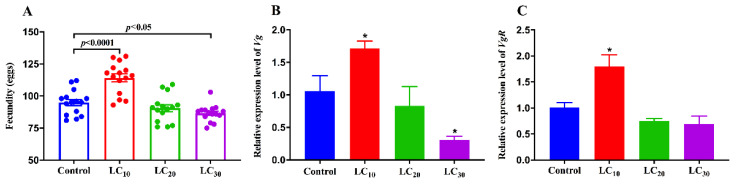
Sublethal effects of imidacloprid on the fecundity of the viruliferous SBPH. (**A**) The number of eggs laid in the viruliferous SBPH exposed to sublethal concentrations of imidacloprid. The transcript levels of *Vg* (**B**) and *VgR* (**C**). Data were analyzed by Student’s *t*-test. The asterisk in (**B**,**C**) indicates significant differences between the treatment and the control (* *p* < 0.05).

The bioassay results of imidacloprid on third instar nymphs of viruliferous SBPH are shown in Table 1. After treatment with the LC_10_ dose of imidacloprid, the fecundity of viruliferous SBPH was significantly increased compared to the control, while the fecundity of SBPH treated by the LC_30_ dose of imidacloprid was significantly lower than the control, and LC_20_ had no obvious effect (Figure 1A).

The effects of imidacloprid on the transcript levels of *Vg* and *VgR* were measured using qRT-PCR. The transcript level of *Vg* was increased significantly by 62% after treatment with LC_10_ of imidacloprid compared with the control, whereas not significantly influenced by LC_20_ of imidacloprid. Treated by LC_30_ of imidacloprid, the transcript level of *Vg* was significantly suppressed by 71% (Figure 1B). The transcript level of *VgR* was significantly up-regulated by 78% by LC_10_, but not significantly affected by the LC_20_ and LC_30_ dose of imidacloprid (Figure 1C).

### 3.2. Effects of Imidacloprid on Apoptosis in Ovaries

**Figure 2:**
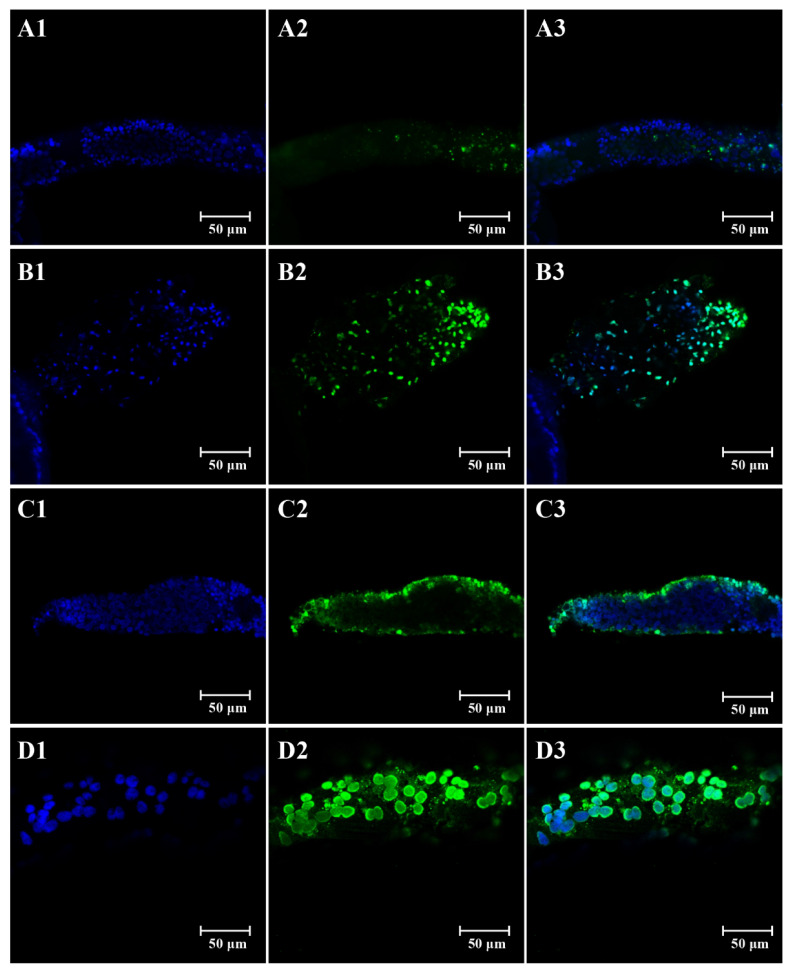
Images of DAPI- and TUNEL-stained SBPH tropharium cells after treatment with LC_10_ of imidacloprid. Morphology of the organization and structure of the developing tropharia from 4-day-old control (**A**) and imidacloprid-treated (**B**) female SBPH, and the aged tropharia from 10-day-old control (**C**) and imidacloprid-treated (**D**) female SBPH. The DAPI- and TUNEL-positive cells were stained with blue (**A1**,**B1**,**C1**,**D1**) and green (**A2**,**B2**,**C2**,**D2**), respectively. (**A1**,**B1**,**C1**,**D1**) and (**A2**,**B2**,**C2**,**D2**) are merged as (**A3**,**B3**,**C3**,**D3**), respectively.

**Figure 3:**
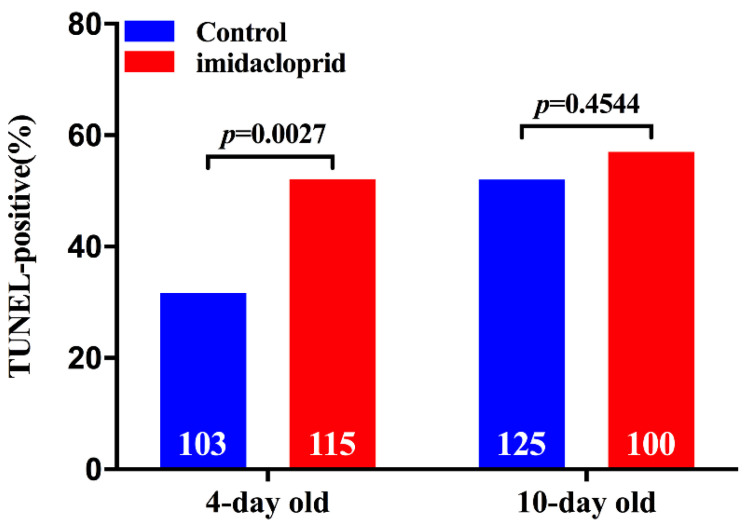
TUNEL quantification. TUNEL-positive tropharia were recorded and presented as a percentage of the examined tropharia both in the control and treatment group of ovaries from viruliferous female SBPH at 4 and 10 days after emergence. The data were analyzed by Chi-square (χ^2^) test. White numbers within bars at the bottom show the total number of examined tropharia.

Apoptotic nurse cells in the tropharium were observed in both control and treated ovaries from viruliferous SBPH at 4 and 10 days after emergence, but the number of apoptotic cells was significantly more in SBPH females treated by imidacloprid than the control (Figure 2). The percentage of tropharia that were TUNEL-positive in SBPH females exposed to imidacloprid (52.8%) (*p* = 0.0055) was significantly higher than the control at 4 days after emergence (31.5%) (Figure 3). The percentage of apoptotic cells had a slight increase at 10 days after emergence, but no significant difference occurred (*p* = 0.5143). The results indicated that exposure to imidacloprid increased the number of nurse cells undergoing apoptosis.

### 3.3. Effects of Imidacloprid on the Expression Levels of Four Caspase Genes in SBPH

**Figure 4:**
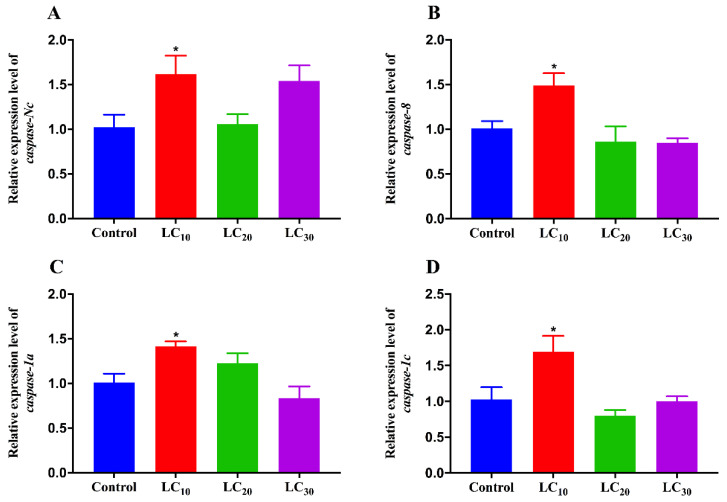
Transcript levels of caspases in the ovaries of viruliferous SBPH treated by sublethal concentrations of imidacloprid. (**A**) *caspase*-*Nc*; (**B**) *caspase*-*8*; (**C**) *caspase*-*1a*; (**D**) *caspase*-*1c*. The asterisk indicates significant differences between the treatment and the control (Student’s *t*-test, * *p* < 0.05).

We measured the transcript levels of four caspase genes via qRT-PCR, and all four caspase genes were significantly increased by LC_10_ of imidacloprid, but not significantly affected by LC_20_ and LC_30_ of imidacloprid compared with the control (Figure 4). The transcript levels of *caspase*-*Nc*, *caspase*-*8*, *caspase*-*1a* and *caspase*-*1c* were up-regulated by 0.58-fold (Figure 4A), 0.48-fold (Figure 4B), 0.40-fold (Figure 4C) and 0.52-fold (Figure 4D) after treatment with LC_10_ of imidacloprid, respectively. The results further demonstrated that sublethal doses of imidacloprid induced apoptosis in the ovaries of SBPH.

### 3.4. Effects of Imidacloprid on RSV Transmission by SBPH

**Figure 5:**
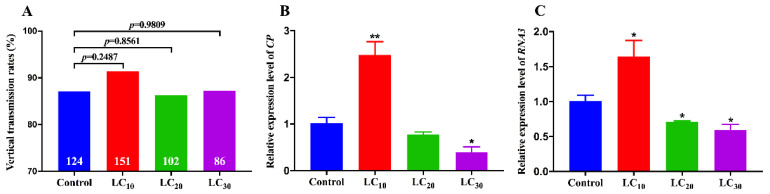
Effects of imidacloprid on RSV transmission of the viruliferous SBPH. (**A**) The vertical transmission rates of RSV in the viruliferous SBPH after treatment with sublethal concentrations of imidacloprid. Relative expression levels of RSV *CP* (**B**) and *RNA3* (**C**) in the ovaries of SBPH. Chi-square (χ^2^) test was used to analyze the data of vertical transmission rates (**A**). The white figure at the bottom of the column shows the total sample size of each treatment. The asterisk in (**B**,**C**) indicates significant differences between the treatment and the control (Student’s *t*-test, * *p* < 0.05, ** *p* < 0.01).

The RSV vertical transmission rate was not increased significantly, whereas partial stimulation was noted with LC_10_ concentration of imidacloprid (91.4%), compared with the control (87.1%) (Figure 5A). Additionally, the transcript levels of *RNA3* and *CP* were significantly up-regulated by LC_10_ of imidacloprid. The expression level of *CP* was increased by 144% after treatment with the LC_10_ dose of imidacloprid (Figure 5B), and the expression level of *RNA3* was increased by 63% compared to the control, respectively (Figure 5C). LC_20_ of imidacloprid did not have effects on *CP* mRNA expressions, though the expression level of *RNA3* was decreased by the LC_20_ dose of imidacloprid compared to the control. Treated by the LC_30_ dose of imidacloprid, the transcript level of *RNA3* and *CP* were both significantly suppressed.

## 4. Discussion

Imidacloprid, a neonicotinoid insecticide, is one of the most widely applied insecticides [20], particularly efficacious against sucking pests such as planthoppers in rice fields [10]. Over time and in the wind, insecticides initially used to kill insects will become sublethal doses, which lead to sublethal effects on insects, including modifying physiological and cellular processes of insects [12,13]. Thus, this study investigated the potential sublethal effects of imidacloprid on SBPH.

Reproduction-related characters are the most essential sublethal parameter studied in the pesticide toxicology of arthropods [52]. Our study showed that, after treatment with the LC_30_ dose of imidacloprid, the fecundity of SBPH was significantly reduced compared to the control. In contrast, the LC_10_ dose of imidacloprid stimulated the reproduction of SBPH. These results are known as hormesis, which is a biphasic dose-response characterized by high-dose inhibition and low-dose stimulation during or following exposure to toxic [53]. This phenomenon has been found in several sucking insects when exposed to sublethal concentrations of imidacloprid, such as *M. persicae* [27,54], *A. glycines* [26], and *Podisus maculiventris* [55]. In *Frankliniella occidentalis* [15], *Cyrtorhinus lividipennis* [28,56], and *Tryporyza incertulas* [57], the fecundity was also stimulated by the lower concentrations of imidacloprid. Additionally, sulfoxaflor [58], triazophos and validamycin [11] could also enhance the fecundity of SBPH with low sublethal concentrations.

We examined the mRNA expressions of *Vg* and *VgR*, and found that *Vg* mRNA expression was significantly up-regulated by the LC_10_ dose of imidacloprid and suppressed by the LC_30_ dose of imidacloprid, which was consistent with the fecundity of SBPH mediated by imidacloprid. Moreover, *VgR* mRNA expression was also significantly increased by the LC_10_ dose of imidacloprid. Previous studies showed that the transcript level of *Vg* and *VgR* was up-regulated in insects exposed to sublethal doses of insecticides. In SBPH, *Vg* and *VgR* mRNA expressions were increased in the females exposed to sublethal doses of triazophos [50]. In *Nilaparvata lugens*, the transcript level of *Vg* was significantly increased by low doses of deltamethrin and triazophos [59]. In *C. lividipennis*, *Vg* mRNA expression was significantly up-regulated by sublethal doses of triazophos, deltamethrin and imidacloprid [28]. These results suggested that *Vg* and *VgR* mRNA expressions partly reflect reproductive changes induced by the sublethal concentrations of insecticides.

The previous study showed that sublethal concentrations of imidacloprid induced the caspase-dependent apoptotic pathway in the honey bees via increasing the expression level of caspase-1 and activating caspase-3 [41]. TUNEL staining showed that the frequency of apoptosis in 4-day-old SBPH ovarioles significantly increased after exposure to LC_10_ of imidacloprid. Meanwhile, we found that four caspase genes were significantly increased by LC_10_ of imidacloprid, which were generally consistent with the increased fecundity, *Vg* and *VgR* mRNA expressions by the LC_10_ dose of imidacloprid. Similar results appeared in the *Wolbachia*-infected SBPH, suggesting that the fecundity of SBPH may be enhanced by increasing the frequency of caspase-dependent apoptosis in the ovaries of infected SBPH [45]. Our findings revealed that sublethal concentration of imidacloprid could induce apoptosis in the ovaries of SBPH, and further suggested that a link may exist between the increased fecundity and apoptosis of SBPH ovaries mediated by sublethal concentrations of imidacloprid.

The virus transmission in insects is closely related to the change of external conditions, including pesticides. The studies demonstrated that pesticides may inhibit the transmission of plant viruses in insects, especially the tomato yellow leaf curl virus (*TYLCV*) transmitted by *B. tabaci*. For instance, sulfoxaflor significantly eliminated the transmission of *TYLCV* [60], and lethal and sublethal doses of flupyradifurone also significantly decreased *TYLCV* transmission in *B. tabaci* [61,62]. However, other studies indicated pesticides could promote virus transmission in insects. Neonicotinoid pesticides clothianidin and imidacloprid promoted the replication of the deformed wing virus in honey bees [63]. Sublethal doses of thiacloprid and imidacloprid induced the higher black queen cell virus (*BQCV*) titers in the honey bees [64,65,66]. Our results indicated that the vertical transmission rate of RSV in SBPH was partly stimulated by the LC_10_ dose of imidacloprid, but no significant differences occurred. However, a recent study has indicated that the vertical transmission rates of RSV were significantly induced by sublethal doses of triazophos in SBPH [50]. The mRNA expression level of *CP* gene or viral *RNA3* segment is used to reflect viral load and has been widely applied to quantify RSV accumulations in SBPH [67]. Thus, our study measured the expression levels of *RNA3* and *CP*, and they were both up-regulated significantly by the LC_10_ dose of imidacloprid, whereas decreased by the LC_30_ dose of imidacloprid. These results are generally consistent with the recent study which showed that after treatment with LC_20_ to LC_50_ of triazophos, the transcript levels of *RNA3* and *CP* were significantly stimulated in the RSV-carrying SBPH [50]. These results showed that low concentrations of insecticides could increase RSV viral load in viruliferous SBPH.

## 5. Conclusions

In conclusion, this study indicated that a sublethal dose of imidacloprid could stimulate the fecundity of SBPH, which may be related to the *Vg* and *VgR* mRNA expressions and the increase of apoptotic cells in ovarioles. Additionally, we found that the LC_10_ dose of imidacloprid stimulated the replication of RSV in SBPH, suggesting that the impact of insecticides on the transmission of viruses by insects should be considered. This study will provide a theoretical basis for understanding the sublethal effects of insecticides and new insights for integrated pest management.

## Figures and Tables

**Table 1:** Toxicity of imidacloprid on the third instar nymphs of viruliferous SBPH.

Insecticide	N	Regression Equation	Dose, 95% Confidence Limits (mg/L)			χ^2^ (*df*)	*p*
			LC_10_	LC_20_	LC_30_		
imidacloprid	540	Y = 1.5256 + 2.2602X	9.34 (7.32–11.90)	14.62 (12.02–17.77)	20.19 (16.95–24.06)	0.770 (3)	0.857

## Data Availability

Data are contained within the article or Supplementary Material.

## Supplementary Materials

The following are available online at https://www.mdpi.com/article/10.3390/insects12121131/s1, Table S1: Primers used for qRT-PCR.
