# Supplementary material for: Sublethal Effects of Imidacloprid on Fecundity, Apoptosis and Virus Transmission in the Small Brown Planthopper Laodelphax striatellus
Source: Insects. 2021 Dec 17;12(12):1131. doi: 10.3390/insects12121131 (PMC8706141; doi:10.3390/insects12121131)
Supplement: Supplementary file 1 [file insects-12-01131-s001.zip › insects-1480638-supplementary.pdf]

Table S1. Primers used for qRT-PCR.

Primer name	Primer Sequence (5’-3’)
Vg-F	CAACATTCTGCCCCAATCCG
Vg-R	TTGGCAGCTCATCAACATCGT
VgR-F	TCACGGAAATCAGCGTCTCTAT
VgR-R	AGGATGAACTAAGGCGTGCTC
caspase-Nc-F	TAATGAGTCACGGCGGAATA
caspase-Nc-R	GCTTGCCCTGCAAGTGTT
caspase-8-F	GAAGAGGAACAACGAACAAGG
caspase-8-R	TGGACAGCTAAGTTGGAGGG
caspase-1a-F	CATGCCAAGGAGACAAACTAGA
caspase-1a-R	CGCCACGAATAGAAACCAG
caspase-1c-F	TGCGTCCAGCGTTGAGAT
caspase-1c-R	CCATTGTGGCAGTAGTCGGT
CP-F	CGCTGGCTGGTATGTCTGTC
CP-R	CCGCTGACTTCTGTTGCTGT
RNA3-F	GCCCAAAACCACACAGACAAA
RNA3-R	CAGTAGTTGTGGGGGGTCTCAT
actin-F	GCGAGAAATCGTCCGAGACAT
actin-R	AACTGGAGGAGGCGGCTGT
